# The Factors Affecting Early Death in Newly Diagnosed APL Patients

**DOI:** 10.1515/med-2019-0074

**Published:** 2019-09-12

**Authors:** Rafiye Ciftciler, Ibrahim Celalettin Haznedaroglu, Salih Aksu, Osman Ozcebe, Nilgun Sayınalp, Umit Yavuz Malkan, Yahya Buyukasık

**Affiliations:** 1 Hacettepe University Faculty of Medicine, Department of Hematology; Ankara, Turkey; 2 Dıskapı Education and Research Hospital, Department of Hematology, Ankara, Turkey

**Keywords:** Acute promyelocytic leukemia, All-trans retinoic acid, Disseminated intravascular coagulation

## Abstract

**Background and aim:**

In the past, acute promyelocytic leukemia (APL) was considered as one of the most rapidly lethal form of acute myeloid leukemia (AML). The objective of this study was to assess clinical parameters affecting early death (ED) in patients with APL.

**Materials and methods:**

Forty-three patients with APL who were diagnosed at Hacettepe University Hospital between the years of 2005 and 2018 were evaluated.

**Results:**

In univariate analyses, presentation with hemorrhage, DIC or infection at diagnosis, ECOG performance score, blast percentage on bone marrow, Sanz score, leukocyte, thrombocyte, fibrinogen and LDH levels were found to be statistically significantly different between patients with ER and patients without ED. In multivariate analysis, presentation with hemorrhage, DIC or infection at diagnosis, ECOG performance score, blast percentage on bone marrow, Sanz score, leukocyte, thrombocyte, fibrinogen, and LDH levels were found to be independent factors that are related with higher rate of ED in 30 days after treatment.

**Conclusion:**

Induction chemotherapy should be started as soon as possible after diagnosis of APL. Improving ED rates may become the greatest challenge for the future treatment of the diseases.

## 1 Introduction

Acute promyelocytic leukemia (APL) is a different clinical subtype of acute myeloid leukemia (AML). In the past, APL was considered as one of the most rapidly lethal forms of AML [1]. More recently, the introduction of all-trans retinoic acid (ATRA) and arsenic trioxide (ATO) has dramatically altered the treatment of APL. APL is now considered a highly curable disease, with 2-year progression-free survival rates of 75-84% [2, 3]. However, APL still has a high incidence of early hemorrhagic complications leading to death mainly due to the presence of coagulopathy, including disseminated intravascular coagulation (DIC), fibrinolysis and proteolysis [4]. Prior to ATRA therapy, early death (ED) related to hemorrhage occurred in up to 26% of cases. However, most clinical trials involving ATRA report ED rates of less than 10% [5]. ATRA is an active metabolite of vitamin A under the family retinoid. Differentiation therapy with ATRA has marked a major advance and become the first choice drug in the treatment of APL. Conversions of 13-cis-retinoic acid and 9-cis-retinoic acid to ATRA is very rapid [6]. Vuletic et al. showed that two major dose and time dependent effects of 13-cis-Retinoic acid (RA) on HL-60 promylocytic cell line. The antiproliferative effect was the first to appear, after only 24 hour of RA treatment, which is followed, after prolonged in vitro incubation, by a prodifferenting effect comprised of accumulation of HL-60 cells in the resting G0/G1 phase of cell cycle and simultaneous increase in CD11b granulocyte differentiation antigen expression [7]. Another study investigated the incidence, treatment, ED rate and long term clinical outcome of APL patients. Twenty two of 41 deaths occurring in 122 APL patients were EDs which were primarily caused by intracranial hemorrhage, DIC, sepsis and multiorgan failure [8]. Contrasting to the life-threatening initial phase of APL, patients surviving this critical period have superior outcomes characterized by low relapse risk and high survival rates than other AML subtypes [9].

Identification of the clinical features and possible risk factors for early mortality trends in APL patients is extremely important for the determination of overall management strategies of the disease course. The objective of this study was to assess clinical parameters affecting ED in patients with APL.

## 2 Materials and methods

### 2.1 Study design and data collection

This study has been performed in a retrospective manner. Forty-three patients with APL who were diagnosed at Hacettepe University Hospital between the years of 2005 and 2018 were evaluated. Demographic data of the patients was obtained from hospital database.

**Ethical approval**: All of the ethical considerations had been strictly followed in accordance with the 1964 Helsinki declaration. As a standard care/action of the hospitals of the Hacettepe Medical School, it has been recognized from the patient records that all of the studied patients had given informed consents at the time of hospitalization and before the administration of chemotherapy and other relevant diagnostic/therapeutic standard of care.

**Informed consent statement**: Informed consent for the procedure was obtained from blood donors for this study.

### 2.2 Patients and disease characteristics

In this study, the patients included were as follows: age ≥18 years, at the time of diagnosis. The diagnostic criteria of APL were based on the World Health Organization Classification of Tumors-Pathology and Genetic of Tumors of Hematopoietic and Lymphoid Tissue [10]. Molecular diagnosis was confirmed by cytogenetic analysis of t (15;17) and/or reverse transcription-polymerase chain reaction (RT-PCR) analysis most of the patients. Leukemic cells were analyzed by flow cytometry and the diagnosis was confirmed by the presence of t (15;17) with fluorescence in situ hybridization (FISH) analysis. RT-PCR allowed detection of two fusion genes (both PML / RARa and RARa / PML) associated with APL. RT-PCR was helpful in detecting PML breakpoints (bcr1-2-3). Acute leukemia flow cytometry was performed at the time of diagnosis for each patients. Peripheral blood mononuclear cells were diluted into a volume of 1.0x10^6^ cells per 100 μl, and labelled with a panel of commercial mouse anti-human monoclonal antibodies in combination with fluorescein isothiocyanate conjugated antibodies [11]. CD33, CD9, CD13, CD117, HLA-DR, CD 34, MPO, CD11b, CD14 monoclonal antibodies were used for flow cytometry. The immune phenotype diagnosis of APL was identified as positive for CD33, CD9, CD13, and CD117 and low expression of HLA-DR and CD34.

DIC was defined according to the International Society on Thrombosis and Haemostasis (ISTH) guidelines using a point scoring system which utilizes prothrombin time (PT), fibrinogen levels and platelet counts to diagnose DIC. A total score ≥5 defined DIC [12].

ED was defined as death because of any cause within 30 days after diagnosis. Clinical features assessed include age, gender, white blood cell count (WBC), platelet count, fibrinogen, creatinine, bone marrow leukemic promyelocyte (BMP) percentage and treatment protocol. Induction therapy consisted of oral ATRA (45 mg/m2 /day) divided into two daily doses, which was maintained until complete hematologic remission and idarubicin (12 mg/m^2^/day) given as an intravenous bolus on days 2, 4, 6 and 8 (AIDA regimen) [13]. Treatment with platelets, fresh frozen plasma, or cryoprecipitate transfusion was provided when bleeding occurred. Prophylactic platelet transfusion strategy was available when the platelet count was less than 50×10^3^/μl. Prophylactic transfusion of fresh frozen plasma (FFP) was predominantly based on fibrinogen level below 100 mg/dL.

### 2.3 Statistical analyses

Statistical analyses were performed using the SPSS software version 25. The variables were investigated using visual (histograms, probability plots) and analytical methods (Kolmogorow-Simirnov/Shapiro-Wilk’s test) to determine whether or not they are normally distributed. Descriptive analyses were presented using means and standard deviations for normally distributed variables. Comparisons were made using the t test, chi-square test, Fisher’s exact test, and analysis of variance. Variables that are found to be significant (p < 0.05) in univariate analysis were tested in multivariate analysis, which was performed using a stepwise logistic regression model. Values of p < 0.05 were considered statistically significant. Survival analyses were made using Kaplan-Meier test.

## 3 Results

### 3.1 Patients characteristics

A total of 43 patients were involved into the study between 2005 and 2018. Patient characteristics are summarized in Table 1. There were 21 males and 22 females with a median age of 40 (range, 18–79) years at the time of diagnosis. The patients were classified with Eastern Cooperative Oncology Group performance status (ECOG PS) 0, 1, 2, 3 and 4 as 10, 22, 6, 3 and 2 respectively [14]. In this study, we found ED of 14 patients (32.6%) in 30 days after the diagnosis APL. Fibrinogen level at diagnosis was statistically significant lower in patients with ED than in patients without ED (p=0.009). No significant difference was found between the two groups in terms of D-dimer level at diagnosis (p=0.16). PT (p<0.001) and aPTT (p=0.01) were statistically significant higher in patients with ED than in patients without ED. In univariate analyses, presentation with hemorrhage, DIC or infection at diagnosis, ECOG PS, blast percentage on bone marrow, Sanz score [15], leukocyte, thrombocyte, fibrinogen, and LDH levels were found to be statistically significant different between patients with ED and patients without ED (Table 2). After univariate analysis, among the factors that had p value <0.05, were taken to multivariate analysis. In multivariate analysis, presentation with hemorrhage, DIC or infection at diagnosis, ECOG performance score, blast percentage on bone marrow, Sanz score, leukocyte, thrombocyte, fibrinogen, and LDH levels were found to be independent factors related with higher rate of ED after starting induction chemotherapy (Table 3).

**Table 1:** Baseline clinical and demographic characteristics of patients

Parameters	Patients with early death in 30 days	Patients without early death in 30 days	Statistical significance
N	14 (32.6%)	29 (67.4%)	
Type of Disease	APL	APL	
Age	37 (18-79)	40 (18-60)	0.65
Gender (male/female)	6/8 (42.9%/57.1%)	15/14 (51.7%/48.3%)	0.58
Treatment type	AIDA regimen	AIDA regimen	
Diagnostic method			
Pathology	14 (100%)	29 (100%)	
Cytogenetic analysis of t(15;17), and/or	13 (92.9%)	28 (96.6%)	
(RT-PCR) analysis			
Fibrinogen at diagnosis (mg/dl) (range)	100 (19-437)	230 (21-525)	0.009
D-dimer (mg/L) (range)	18.5 (0.3-40.0)	5.2 (0.3-40.0)	0.16
aPTT (second) (range)	31 (23.0-63.5)	27 (14.8-34.1)	0.01
PT (INR) (range)	1.6 (1.1-4.0)	1.2 (1.0-1.7)	<0.001

Abbrevations: aPTT: Active partial thromboplastin time; N: Number; INR: International normalized ratio

**Table 2:** Univariate analysis of parameters affecting early death in 30 days after diagnosis

Parameters	Patients with early death in 30 days	Patients without early death in 30 days	Statistical significance
Presentation with hemorrhage at diagnosis	7 (50%)	4 (13.8%)	0.01
Presentation with DIC at diagnosis	11 (78.6%)	9 (31.0%)	0.003
Presentation with infection at diagnosis	13 (92.9%)	15 (51.7%)	0.008
ECOG PS			0.01
ECOG PS 0	1 (7.1%)	9 (31.0%)	
ECOG PS 1	6 (42.9%)	16 (55.2%)	
ECOG PS 2	2 (14.3%)	4 (13.8%)	
ECOG PS 3	3 (21.4%)	0	
ECOG PS 4	2 (14.3%)	0	
Blast percentage at diagnosis	80 (50-90)	35 (15-90)	0.005
Hemoglobin at diagnosis (gr/dl) (range)	8,3 (4-13,4)	9.6 (6.1-15.6)	0.46
Leukocyte at diagnosis (range)	31.7×10^3^/μl (0.7 -200×103^/^μl)	1.5×10^3^/μl (0.4-87.5×10^3^/μl)	<0.001
Thrombocyte at diagnosis (range)	19×10^3^/μl (5-61×10^3^/μl)	40×10^3^/μl (11-101 1×10^3^/μl)	0.40
Creatinine at diagnosis (mg/dl) (range)	0.81 (0.49-1.67)	0.77 (0.44-9.0)	0.57
Fibrinogen at diagnosis (mg/dl) (range)	100 (19-437)	230 (21-525)	0.009
CRP at diagnosis (mg/dl) (range)	6.6 (0.7-26.3)	2.8 (0.2-45.3)	0.53
LDH at diagnosis (u/lt) (range)	1230 (370-7200)	430 (200-4200)	0.01
Sanz score			0.001
Low	1 (7.1%)	10 (34.5%)	
Intermediate	3 (21.4%)	15 (51.7%)	
High	10 (71.4%)	4 (13.8%)	

Note: Categorical and continuous data were compared by the Chi-square (or Fisher’s Exact test if required by sample size) and Independent-samples T-test, respectively. Bivariate correlation analysis for categorical variables was done by Spearman’s correlation analysis.

### 3.2 Overall Outcomes

**Figure 1:**
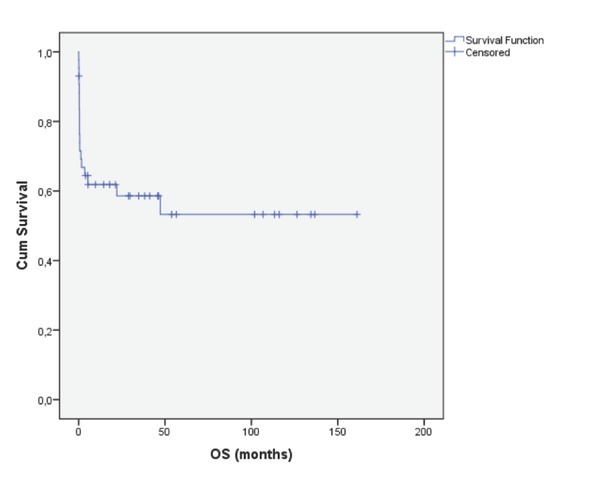
Overall survival of APL patients

Of the 43 patients, the median follow-up period was 14 months (range, 0.07-161) for the entire group (figure 1). The 3-year OS for patients who had Sanz score low, intermediate and high were 72%, 68% and 19%, respectively (p=0.001). The 5-year OS for patients who had Sanz score low and intermediate were 72% and 68%, respectively.

**Figure 2:**
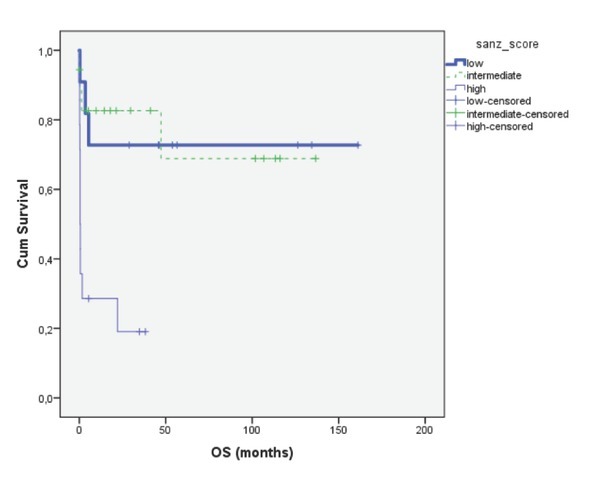
Overall survival of the patients according to Sanz score (p=0.001)

## 4 Discussion

APL is now the most curable subtype of AML. The primary cause of treatment failure in patients with APL is ED, defined as death within the first 30 days after diagnosis. Early death rate needs to be improved in management of APL. During the last 2 decades, the treatment of APL has gained remarkable improvements due to the induction of ATRA and ATO, improved diagnostic tools and molecular monitoring, improved supportive care and reduced doses of chemotherapy [1, 16].

ED rates range from 7% to 14% in APL patients, therefore, ED remains a serious problem in those patients [13, 17]. In this study, ED rate was found to be 32.6% (14/43 patients). This study showed Sanz score, ECOG PS, presentation with DIC, hemorrhage or infection, blast percentage on bone marrow biopsy, WBC count, fibrinogen and LDH level at diagnosis as predictor factors for ED. However, in this study age was not found as a predictor factor for ED. The ECOG performance score was an independent factor affecting early death. This effect of ECOG score is regardless of age. So it may be interpreted that performance of patients is not always parallel with age, elder patients may also have good performance.

Previous population-based studies reported performance status, leucocyte count and age to be risk factors for ED in APL patients [9, 18, 19]. In past studies the Sanz score was a well-established prognostic score in APL patients [15]. Additionally, ED are mostly attributable to the presenting coagulopathy [20]. Risk factors for severe hemorrhage have been reported to be low initial fibrinogen level and performance status, also high WBC counts in APL patients [4]. Another study showed that high-risk APL patients had leucocytes (>10×10^9^/L), which is an important predictive factor for ED during induction chemotherapy [21]. A recent study has shown that higher peripheral WBC counts are closely related to early ED in APL patients and control of peripheral WBC count may reduce the ED rate of APL patients [22]. Additionally, LDH within many biochemical parameters demonstrates a very valuable enzyme in patients with malignancy with possibility for easy routine measurement in many clinical laboratories. The new investigations showed that LDH based on the principle that tumor cells release intracellular enzymes trough damaged cell membrane that is mostly consequence in intracellular mitochondrial machinery alteration and apoptosis deregulation. It is showed that it is very useful to analyzed intracellular LDH activity in different cell line and tumor tissues obtained from patients, not only to understanding complexity in cancer biochemistry but also in early clinical diagnosis [23].

In this study, we evaluated whether different parameters such as age, gender, hemorrhage, DIC or infection at presentation, blast percentage, hemoglobin level, WBC count, thrombocyte count, fibrinogen level, creatinine level, LDH level, Sanz score, ECOG PS in APL patients predicted ED during and after inducton chemotherapy with in the first 30 days. Sanz score was a predictive factor for early death in APL patients. LDH was significantly higher in both univariate and multivariate analysis in patients who died within the first 30 days in this study. Multivariate analysis showed that WBC count was the most important prognostic factor in APL among laboratory analysis. Infection, DIC or hemorrhage at the time of diagnosis of the patients were determined as parameters predicting ED.

No significant differences was found in hemoglobin, creatinine, thrombocyte and CRP levels between patients with ED and patients without ED in univariate analyses. As expected, OS was longer in patients who had low Sanz score than patients who had high Sanz score. While therapy for APL has improved dramatically over three decades, our results demonstrated that ED still remains a significant problem.

Our study had some limitations. First, this study was retrospective. Second all patients were not completed induction chemotherapy protocol. Two patients died 2 days after the onset of induction chemotherapy and 2 patients died 4 days after the onset of induction chemotherapy. Third fewer patients included in this study. The utilization of more aggressive supportive measures in patients with a high risk of ED needs to be tested prospectively. Additionally, it is still unclear whether further refinements of APL therapy (e.g. the addition of arsenic trioxide to the induction regimen) would decrease ED rates in this otherwise highly curable disease [24].

In conclusion, if necessary aggressive prophylactic transfusion to maintain high platelet and fibrinogen transfusion thresholds could reduce hemorrhage in APL patients. Infection should be controlled in patients with newly diagnosed APL. Induction chemotherapy should started as soon as possible after diagnosis as APL. Improving early death rates may become the greatest challenge for the future treatment of APL.
